# Characterization of *Pestivirus scrofae* infection in the tissues of a persistently infected boar

**DOI:** 10.1186/s40813-026-00514-4

**Published:** 2026-06-16

**Authors:** Lilla Dénes, Lukas Schwarz, René Brunthaler, Sandra Högler, Gyula Balka

**Affiliations:** 1 Department of Pathology, University of Veterinary Medicine, Budapest, Hungary; 2 National Laboratory of Infectious Animal Diseases, Antimicrobial Resistance, Veterinary Public Health and Food Chain Safety, University of Veterinary Medicine, Budapest, Hungary; 3 Clinical Centre for Population Medicine in Fish, Pig and Poultry, Clinical Department for Farm Animals and Food System Science, University of Veterinary Medicine Vienna, Vienna, Austria; 4 Pathology, Centre of Pathobiology, Department for Biological Sciences and Pathobiology, University of Veterinary Medicine Vienna, Vienna, Austria

**Keywords:** Atypical porcine pestivirus, Testicular infection, Tissue tropism, Persistent infection

## Abstract

**Background:**

*Pestivirus scrofae* (previously atypical porcine pestivirus, APPV) is a member of the genus *Pestivirus* within the family *Flaviviridae*. This virus has been established as the etiological agent of congenital tremor (CT) type AII, a neurological disorder in newborn piglets characterized by generalized tremors. Despite this association, the epidemiological characteristics of the virus, including its transmission dynamics and patterns of spread between herds, remain poorly understood. It has already been confirmed that, similarly to other flaviviruses, APPV can be shed by semen. Previously, we have identified interstitial Leydig cells, the peritubular myoid cells and smooth muscle cells of medium-sized arteries as target cells of APPV in the testicles of newborn, congenitally infected, CT-affected piglets.

**Results:**

In our present study, we examined FFPE tissue samples obtained from a persistently infected 6-months-old, sexually mature boar, born with CT, that was still shedding 2.1 × 10^9^ GE/mL APPV in its semen. We detected viral genome by RNAscope in situ hybridization method in the T-cell zones of the lymph nodes draining the reproductive organs and the GALT of the ileum, periarteriolar lymphoid sheaths of the spleen and in cells of the intestinal crypts in the ileum. We also identified positive cells in the molecular, granular and the Purkinje-cell layer of the cerebellum, and in the neurons of the spinal cord and the cerebral cortex. We found weak positive signal in the colon, but none in the lungs and the liver. Interestingly, positive signals were detected in the outer layers of the adrenal gland cortex (*zona glomerulosa* and *fasciculata)*, as well as in the acinar cells of the exocrine pancreas. In reproductive organs, viral nucleic acid was detected in the Leydig cells and peritubular myoid cells of the testicles, in cells within the seminiferous tubules and in the epididymis. We also found positive cells in the prostate and the bulbourethral gland. The shedding of the virus in semen supports the notion that infected cells within the reproductive tract may serve as a source of the virus in semen. These affected cells of the reproductive tract could serve as source of the virus in semen.

**Conclusion:**

Our results suggest that APPV is capable of crossing the blood–testis barrier, and may be associated with persistent infection, including cases likely originating from congenital exposure, in pigs and boars that can actively shed the virus. Therefore, the use of such animals for breeding purposes should be approached with caution. Further research is needed to elucidate the underlying mechanisms of persistent infection.

## Introduction

Atypical porcine pestivirus (APPV, species *Pestivirus scrofae*) is a member of the *Pestivirus* genus within the family *Flaviviridae* [1]. The genus comprises 19 species, six of which, the *Pestivirus bovis* (bovine viral diarrhea virus 1), *Pestivirus tauri* (bovine viral diarrhea virus 2), *Pestivirus suis* (classical swine fever virus), *Pestivirus australiaense* (Bungowannah virus), *Pestivirus scrofae* and *Pestivirus L* (Linda virus) have been described in swine [2–4].

APPV was first identified in the United States in 2015 [5]. Since then, the virus has been reported in commercial pig farms across several European countries [6–18], in China [19] and in Brazil [20]. The virus has also been identified in wild boar populations in Germany and Serbia [21], Spain [22], Italy [23], South Korea [24], and Sweden [25], indicating that wild boars may function as a reservoir host, analogous to the role described for *Pestivirus suis.*

Members of the *Flaviviridae* family, including the species within the *Pestivirus* genus, are known for their ability to cause transplacental infection, leading to neuronal damage and developmental defects such as cerebellar hypoplasia, demyelination, or, in severe cases, stillbirth. The resulting neurological impairment may manifest as congenital tremor (CT), which is observed as tremors of the head and limbs in neonatal piglets, and is often complicated with splayed legs [3, 12, 26, 27]. CT is classified based on the absence (type B) or presence (type A) of lesions in the central nervous system [26, 28]. Specifically, CT type A-II is attributed to APPV infection and is associated with hypomyelination in both the cerebellum and spinal cord [13, 29, 30].

Apart from the central nervous system, the virus has been found in a range of other tissues, including lymphoid organs such as the thymus, lymph nodes, tonsils, and spleen, as well as in the digestive tract encompassing the small and large intestines, pancreas, and liver, in addition to the lungs and heart [6, 27]. APPV has also been observed in endothelial cells, vascular tunics, fibroblasts, and the fibromuscular stroma of multiple organs, including the liver, kidney, colon, lung, lymph nodes, tonsils, thymus, and thyroid [31, 32]. Notably, viral RNA has been detected in the testis in the tunica albuginea and within the lumen of the seminiferous tubules as well [32].

Furthermore the detection of APPV in boar semen and preputial fluids [12, 13, 33–35] indicates that the male reproductive tract may serve as a reservoir for the virus, as has been observed for other pestiviruses like *Pestivirus suis* [36] and *Pestivirus bovis/tauri* [37], thereby supporting viral persistence and spread. Since sperm cells are considered immunologically foreign, the body must prevent the immune system from attacking them. This protection is primarily ensured by the blood-testis barrier, which is created through tight junctions between Sertoli cells, interactions among various testicular cell types, and the activity of immunoregulatory molecules. The testes must carefully coordinate both branches of the immune system: the innate immune response, which is fast and non-specific, and the adaptive response, which is slower but antigen-specific, involving B and T lymphocytes. While it is crucial to shield developing germ cells, the testicular environment also needs to respond effectively to infections, as these can trigger inflammation and immune reactions that may damage the sperm [38].

Our research group found high prevalence of APPV in processing fluid samples collected from Hungarian pig herds which led us to investigate the cellular targets of the virus in the testicles of newborn piglets affected by CT. Using RNAscope in situ hybridization and immunohistochemistry on consecutive slides, we identified the target cells of APPV in the testicle of newborn CT-affected piglets: interstitial Leydig cells, peritubular myoid cells and endothelial smooth muscle cells of medium-sized arteries [39]. In that study we did not find viral genome beyond the Blood-Testis barrier (i.e. in Sertoli cells and germ cells), unlike what has been described in the case of bovine viral diarrhea virus (BVDV) where persistently infected bulls were shedding the virus through semen for a prolonged period. The authors showed that the virus was localized in the seminiferous tubules of the animals [40] similarly to the Zika virus of the *Flavivirus* genus [41], which is also known for being transmitted sexually. Persistent infection is an important feature of closely related BVDV that plays an important role in the spread of the virus.

Schwarz et al. (2017) followed the course of viraemia of CT affected piglets for 6 months and found high levels of APPV genome (2.1 × 10^9^ GE/mL) in the semen and saliva of a boar that reached sexual maturity by that time, which was clinically recovered from CT. Our aim was to identify the target cells in the archived organs of the animal with special focus on its reproductive organs, and to compare the results to what we found in newborn animals with CT.

## Materials and methods

### Samples

We examined tissue samples (colon, spleen, ileum, liver, testicles, lungs, adrenal glands, bulbourethral gland, prostate, pancreas, cerebellum, cerebral cortex and spinal cord) in formalin-fixed paraffin-embedded (FFPE) blocks obtained from a 6-month-old male pig that was born with congenital tremor (CT), later became symptom-free, and was followed until sexual maturity at the University of Veterinary Medicine Vienna. At 6 months of age, the animal still shed a significant amount of APPV in its semen (2.1 × 10^9 GE/mL) and saliva (2.9 × 10^9 GE/mL), while the virus was detected at a significantly lower level in the serum (2.0 × 10^7 GE/mL). APPV NS3 helicase-specific antibodies were detectable in the animal from birth until 8 weeks of age [13], indicating that the animal was already seronegative at the time of sampling.

### RNA extraction

We extracted RNA from the FFPE tissue samples using the AllPrep DNA/RNA FFPE Kit (Qiagen, Hilden, Germany) following the manufacturer’s instructions. Briefly, we sliced off the superficial, oxidized layer from the blocks and prepared two 10 μm thick sections per block using a rotary microtome (Leica Biosystems, Deer Park, USA). We placed the sections in sterile collection tubes and immediately vortexed them in 1 ml of xylene for 15 s, followed by centrifugation (2 min, 12,000 rpm). We carefully pipetted off the supernatant without disturbing the sediment and then added 1 ml of absolute ethanol to remove any remaining xylene. After vortexing and centrifuging again, we pipetted off the supernatant. We placed the collection tubes containing the pellets in a preheated thermostat at 37 °C with an open lid and incubated them for 10 min or until the ethanol smell was no longer noticeable. Then, we added 150 µl of proteinase K buffer and 10 µl of proteinase K solution to the samples. After vortexing for 15 s, we incubated them at 56 °C for 15 min. To enhance efficient precipitation, we placed the samples on ice for 3 min and then centrifuged them at 16,000 rpm for 15 min. From this point on, we followed the complete RNA extraction protocol optimized in the kit’s user manual. After centrifugation, we carefully pipetted off the supernatant, transferred it to a new sterile collection tube, and incubated it at 80 °C for 15 min. Subsequently, nucleic acid purification was carried out using a silicon membrane-based column (RNeasy MinElute, Qiagen, Hilden, Germany). The nucleic acid extracted from the FFPE samples was always suspended in 60 µl of elution buffer (EB) (Qiagen, Hilden, Germany). Subsequently, it was either subjected to RT-qPCR analysis immediately or stored at − 80 °C until further testing.

### Quantitative reverese-transcription PCR (qRT-PCR)

qRT-PCR on serum, saliva and semen samples was performed by Schwarz et al. (2017) [13] at the University of Veterinary Medicine Vienna using primers (P1: 5′-AGTTCAGAAAT CCGGTAGCTG-3′ and P2: 5′-CTACCAGCCTGA GGTCTTC-3′) and a TaqMan probe (P3: 5′-FAM-GTTTCGACACCAAAGCTTGGG ACACTCA-TAMRA-3′), specifically designed for the detection of the NS5b protein coding region of APPV.

For the detection of the APPV genome in the FFPE samples we applied a multiplex TaqMan probe-based system, described by F. Yuan et al. (2022) [42], which utilizes primers and fluorescence-labeled probes designed for the NS3 and NS5b regions (NS3-F2 Q: GTGGTCATAGAYACYATGCAG, NS3-R2 Q: TTCCTCTGGCCCTGTTCTTC, NS3-P2 Q: FAM-TAGTGAATTTCTCVGCAAAGATGCC-BHQ1; NS5B-F Q: TCGTCACTTAYAAGAAACCACG, NS5B-R Q: TTTACCCACTTGTACATTATTTTGGT, NS5B-P Q: FAM-ATACAGTACCCTGAGGCAGTCAC-BHQ1), which was proven to be suitable for the detection of all variants we have identified in Hungary so far [8, 16]. QRT-PCR was conducted using the One Step RT-PCR Kit (Qiagen, Hilden, Germany) in a 25 µl reaction mixture, which consisted of 7.5 µl of RNase-free water, 5 µl of 5x QIAGEN OneStep RT-PCR buffer, 1 µl of 10 mM dNTP, 2.5 µM final concentration of each primer, 1.25 µM final concentration of each probe, 0.1 µl of RI (Ribolock, 40U/µl, Fermentas, Waltham, USA) 1 µl of QIAGEN OneStep RT-PCR Enzyme Mix, and 5 µl of template DNA. The thermal cycling protocol included reverse transcription at 50 °C for 40 min, inactivation of the reverse transcriptase enzyme, and heat activation of Taq polymerase at 95 °C for 15 min. This was followed by 45 cycles of denaturation at 95 °C for 15 s, primer annealing, and elongation at 58 °C for 30 s. Fluorescence data were collected during the final step of the cycling process at an emission wavelength of 510 ± 5 nm, corresponding to the FAM (6-carboxyfluorescein) dye, using the green channel of a Rotor Gene Q (Qiagen, Hilden, Germany) instrument.

### RNA in situ hybridization (RNAscope)

Manual RNA in situ hybridization (ISH) targeting the APPV genome in various tissues was conducted using the RNAscope^®^ 2.5 HD Detection Kit (RED) (Advanced Cell Diagnostics, Newark, CA, USA) in accordance with the manufacturer’s instructions. A mixture of Z-shaped probe pairs (catalog number: 503381) designed to target nucleotides 1–2816 of the genes encoding the NS2–NS3 proteins was employed.

In brief, 5 μm-thick sections were prepared from FFPE blocks and subjected to pretreatment involving heat and protease treatment prior to hybridization with the target oligonucleotide probe. Following hybridization, preamplifier, amplifier, and AP-labeled oligos were applied sequentially, and chromogenic precipitate was subsequently developed. To verify RNA integrity and reaction specificity, a positive control probe targeting Ss-PPIB RNA (*Sus scrofa* peptidylprolyl isomerase B, catalog number: 428591) and a negative control probe targeting bacterial dapB RNA (*Bacillus subtilis* dihydrodipicolinate reductase, catalog number: 310043) were included.

Counterstaining was performed with Hematoxylin GILL II (Merck, Darmstadt, Germany) for 60 s. Slides were then dried at 60 ˚C for 10 min, followed by immersion in xylene 2–3 times prior to mounting.

## Results

**Fig. 1:**
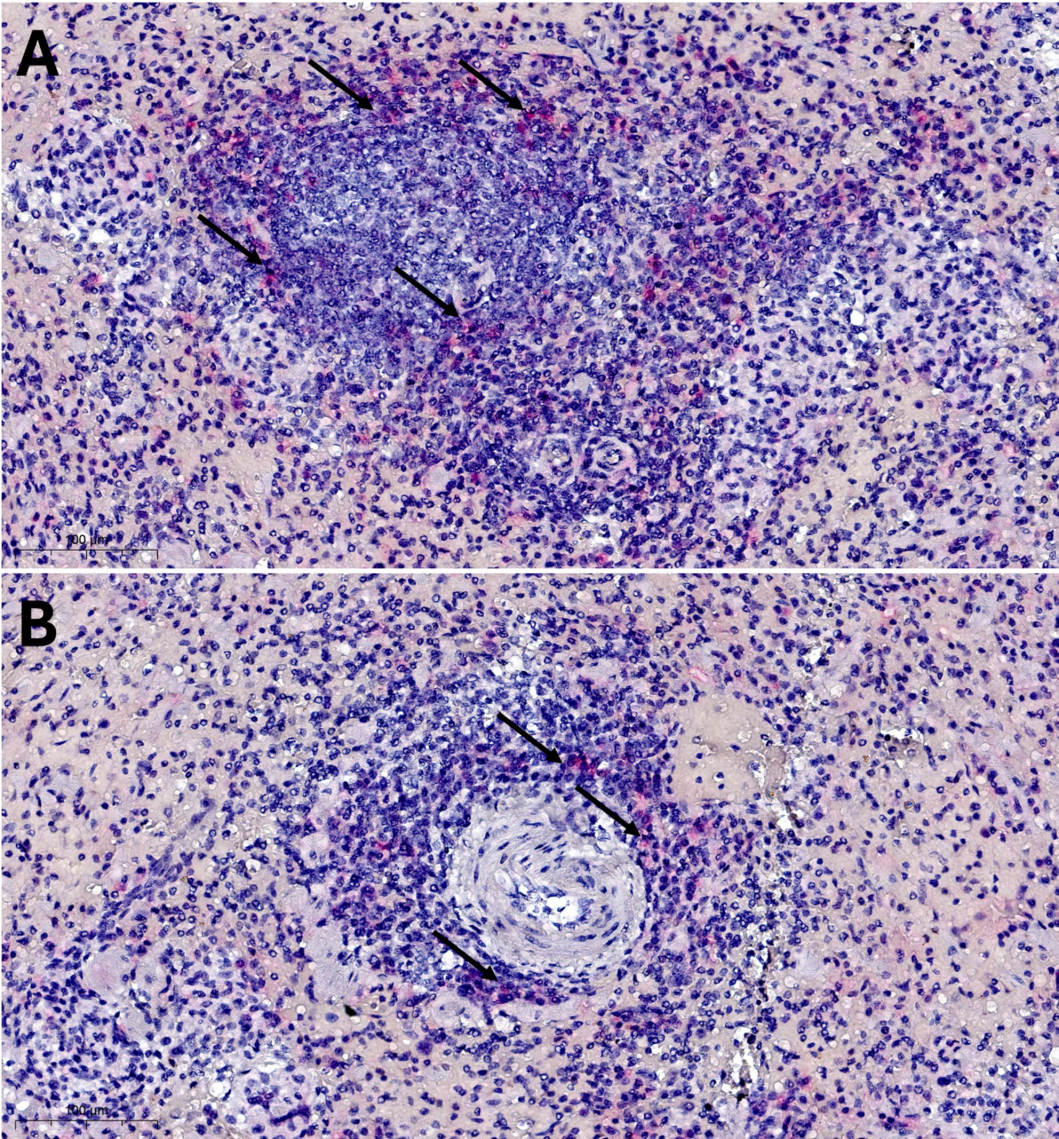
Identification of APPV using the RNAscope method in the infected spleen. Black arrows point to the marginal zone of the follicle (**A**) and the periarteriolar lymphoid sheath (PALS, **B**). ISH, 40×, Scale Bar = 100 μm

We examined the organs of a boar obtained from a 2017 study conducted at the University of Veterinary Medicine Vienna using the RNAscope method for the detection of APPV. In the spleen positive cells were identified in the follicles (Fig. 1A), that are predominantly composed of B lymphocytes, and in the periarteriolar lymphoid sheaths formed by T lymphocytes (Fig. 1B).

**Fig. 2:**
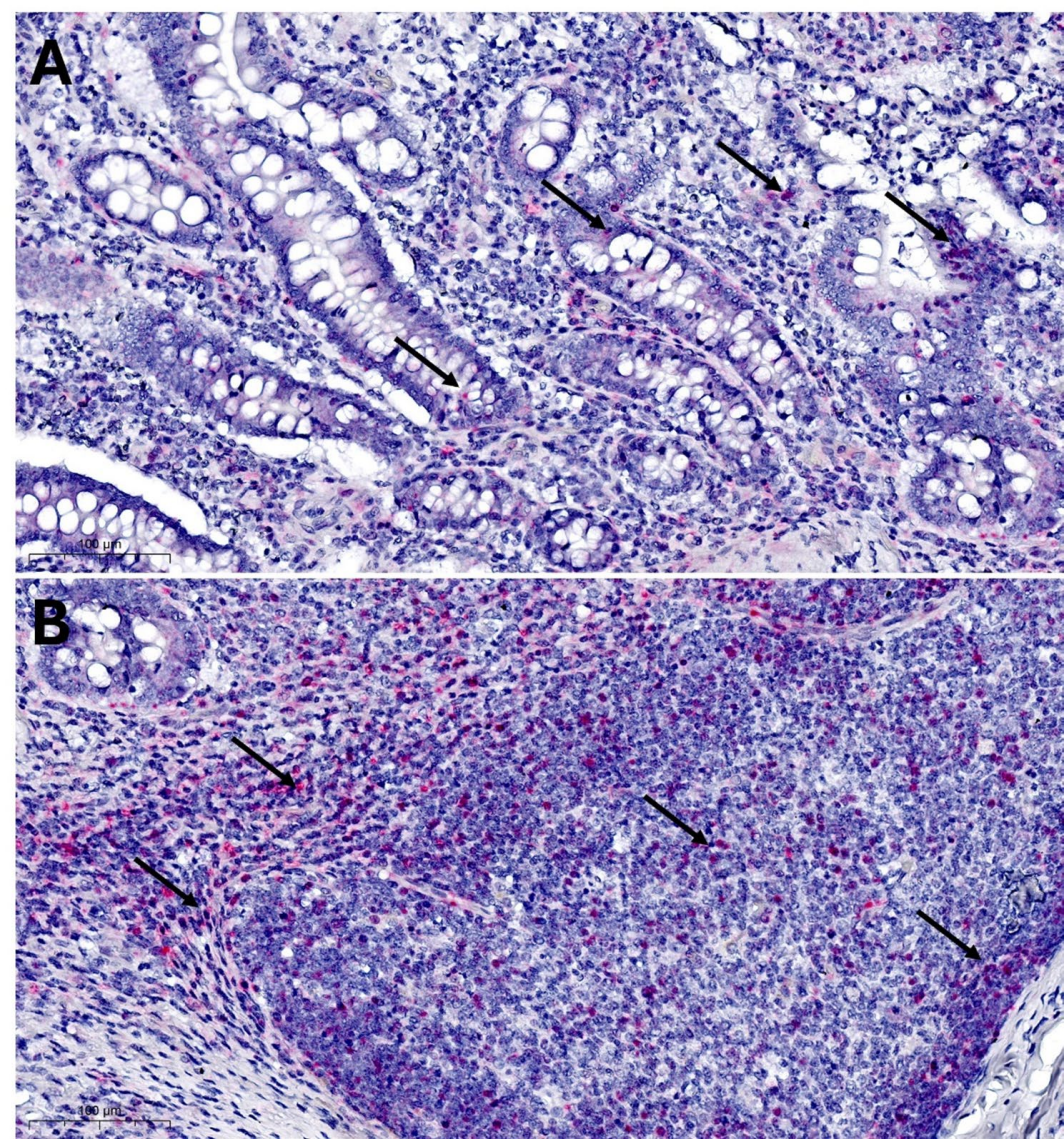
Identification of APPV using the RNAscope method in the infected ileum. Black arrows indicate the positive signal in the intestinal crypts (**A**) and the Gut Associated Lymphoid Tissue (GALT, **B**). ISH, 40×, Scale Bar = 100 μm

Positive signal was also detected in cells of the intestinal crypts (Fig. 2A) and in the Gut Associated Lymphoid Tissue (GALT, 2B) region of the ileum.

**Fig. 3:**
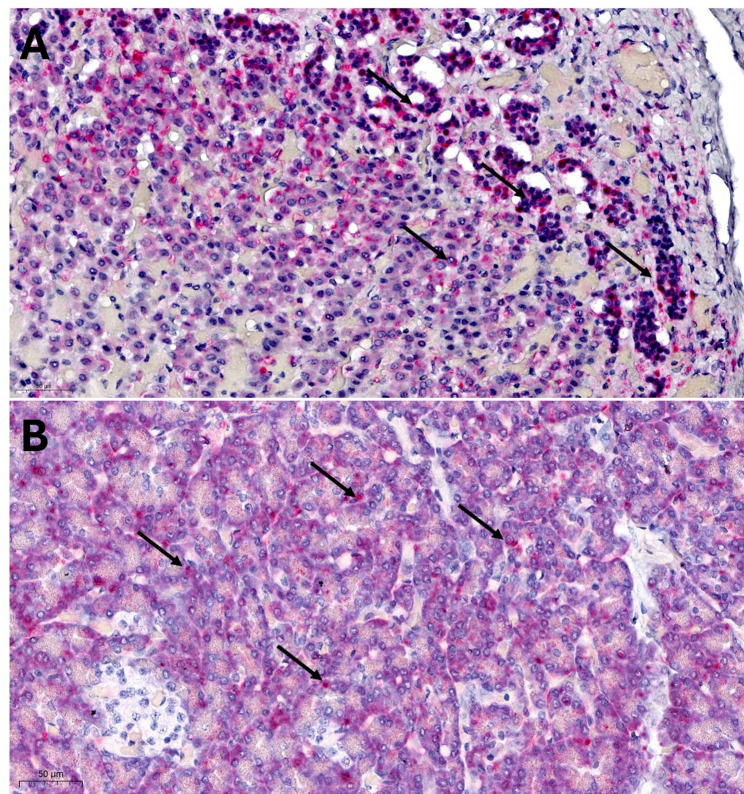
Identification of APPV using RNA in situ hybridization (RNAscope) in the adrenal gland (**A**) and pancreas (**B**) of an infected 6-month-old boar. Positive reactions are represented by red precipitates. Black arrows indicate positive cells in the *glomerulosa* and *fasciculata* (**A**) and the acinar cells (**B**). ISH; 30× (**A**) and 50× (**B**), Scale Bar = 50 μm

Positive signals were detected in the outer layers of the adrenal gland cortex (*zona glomerulosa* and *fasciculata*, Fig. 3A), as well as in the acinar cells of the exocrine pancreas (Fig. 3B).

**Fig. 4:**
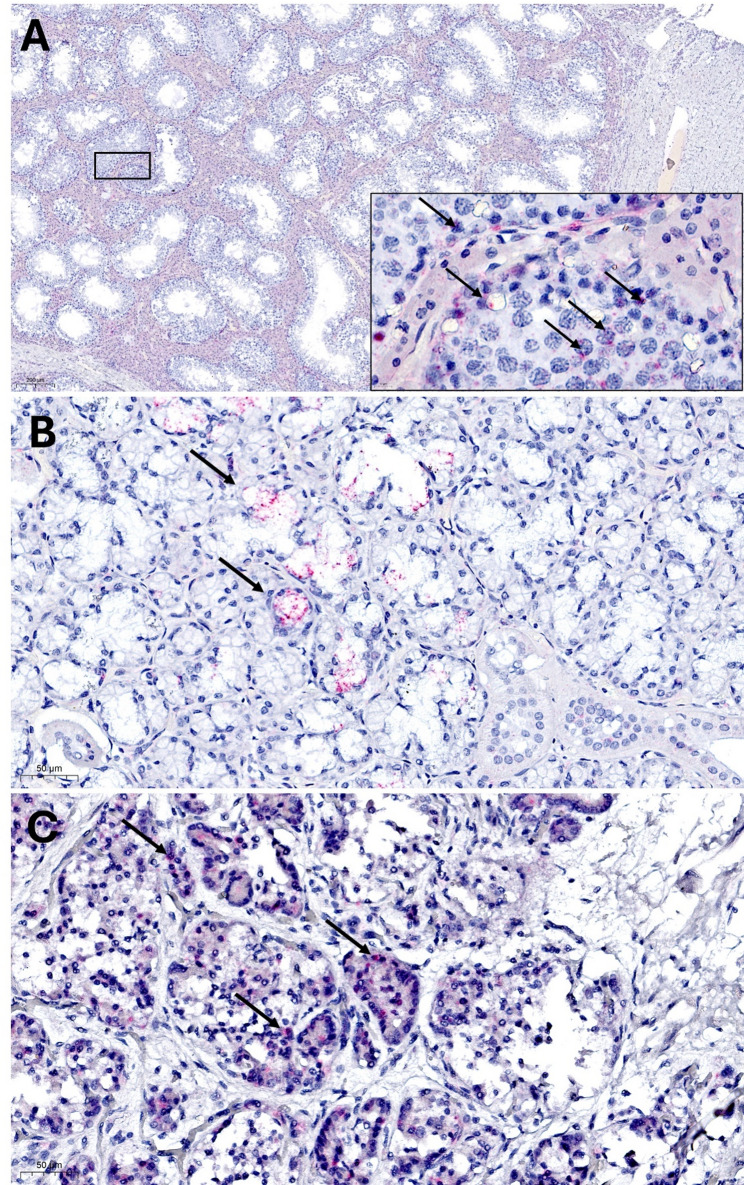
Identification of APPV by RNA in situ hybridization (RNAscope) in the testicular tissue (**A**), bulbourethral gland (**B**), and prostate (**C**) of an infected 6-month-old boar. Black arrows indicate the positive cells in the interstitium, in the peritubular area and within the seminiferous tubules (A inset), in the bulbourethral gland (**B**) and in acinar cells of the prostate (**C**). ISH, 5× (**A**), 90× (A inset), 60× (**B**, **C**). The scale bar = 200 μm (**A**), 10 μm (A inset), 50 μm (**B**, **C**)

Strong signal intensity was observed in the reproductive organs (Fig. 4) as well. In case of testicular tissue, we identified APPV-positive signals in Leydig cells located in the interstitial region, in peritubular myoid cells, and in cells located beyond the blood-testis barrier (Fig. 4A). In addition to this, prominent staining was observed in the bulbourethral gland (Fig. 4B) and the acinar cells of the prostate (Fig. 4C).

**Fig. 5:**
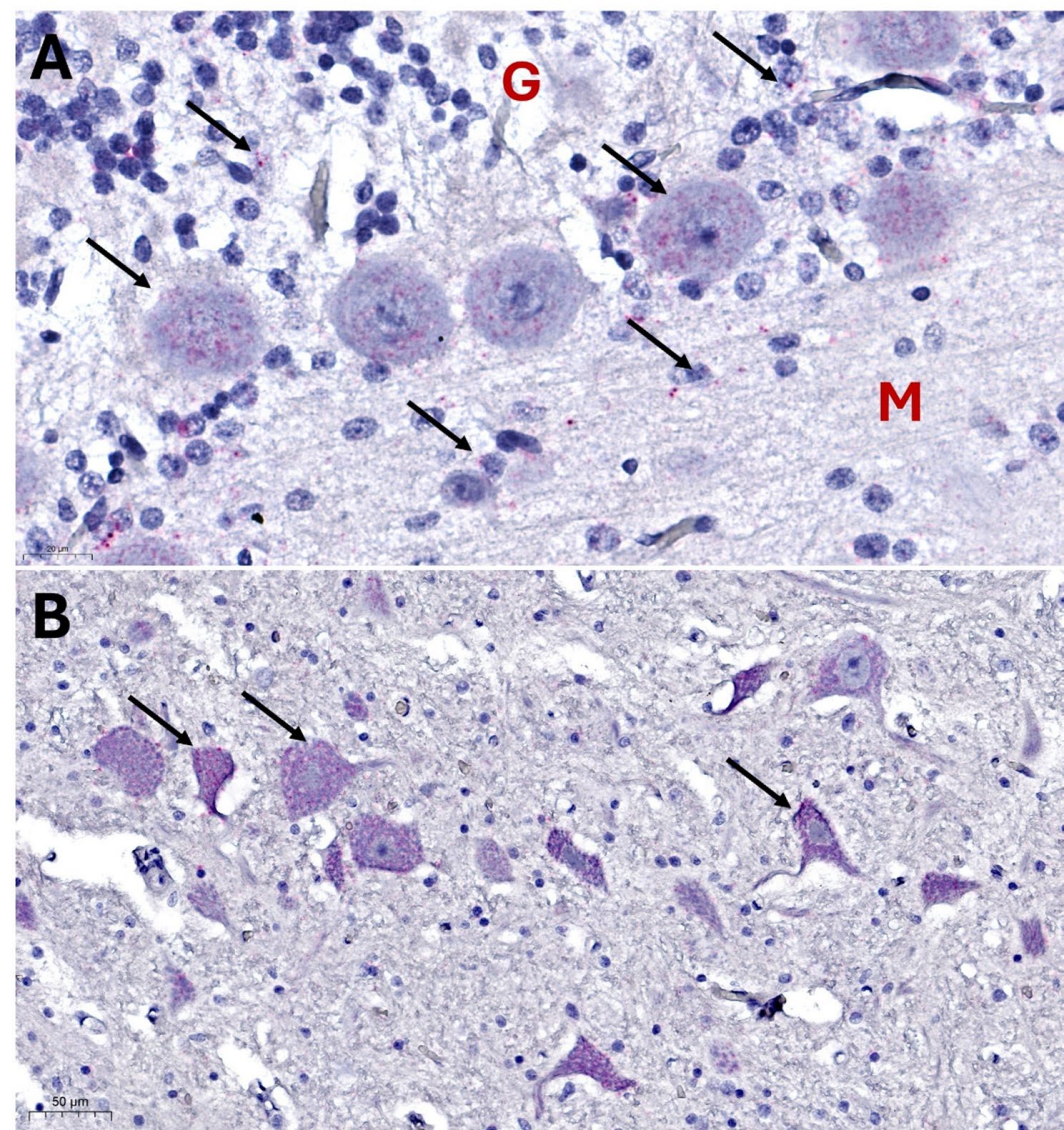
Identification of APPV using the RNAscope method in the cerebellum (**A**) and spinal cord (**B**) of an infected 6-month-old male pig. Positive reactions are represented by red precipitates. Black arrows indicate positive cells in the molecular, granular and Purkinje layer (**A**) and spinal motor neurons (**B**). The granular cell layer is marked with ‘G,’ and the molecular cell layer with ‘M.’ ISH, 63× (**A**), 60× (**B**), Scale bar = 20 μm (**A**) and 50 μm (**B**)

We observed weak staining in the cerebellum of the animal, mostly in the layer of the Purkinje cells (Fig. 5A). Furthermore, we identified APPV-infected neurons in the gray matter of the spinal cord (Fig. 5B) and the cerebral cortex, while no positive cells were identified in the brainstem.

We also performed RNA extraction, and RT-qPCR (Table 1) from the archived FFPE tissue samples used for RNAscope ISH to further validate the presence of APPV nucleic acid in our samples. In the case of the adrenal cortex, cerebral cortex and spinal cord, only a very small amount of virus genome was detected using both the RNAscope and qRT-PCR methods.

**Table 1:** Results of APPV specific qRT-PCR and RNAscope performed on the tissue samples from the 6-month-old male pig. The symbols “+” and “–“ represent a positive or no signal, respectively

Organs	qRT-PCR (Cq)	RNAscope
spleen	33	+
lymph nodes	32	+
ileum	32	+
colon	38	+
adrenal gland	38	+
pancreas	34	+
testis	29	+
bulbourethral gland	33	+
prostate	34	+
cerebellar cortex	35	+
spinal cord	40	+
cerebral cortex	40	+
lungs	–	–
liver	–	–

## Discussion

Our study demonstrates the presence of *Pestivirus scrofae* (formerly atypical porcine pestivirus, APPV) within both somatic and germinal compartments of the testis in a sexually mature boar, including cells beyond the Sertoli cell barrier. This finding expands our understanding of the tissue tropism and transmission potential of the virus and marks the first direct histological evidence of its localization within germ cells in an adult, virus-shedding boar. Our results provide a plausible explanation regarding the source of APPV genome in boar semen that has been widely reported previously [13, 33–35].

In contrast to our earlier observations in CT-affected newborn piglets, where viral genome was detected only in interstitial Leydig cells, peritubular myoid cells, and smooth muscle cells of testicular arteries [39], the current study reveals viral nucleic acid within Sertoli cells and germ cells beyond the blood-testis barrier. This barrier is a critical component of testicular immune privilege, formed by tight junctions between Sertoli cells, and functions to shield developing germ cells from systemic immune surveillance [38]. Crossing this barrier implies either a change in the susceptibility of the testicular microenvironment during sexual maturation or an adaptive strategy of the virus that allows for long-term persistence and potential transmission.

The localization of APPV in immune-privileged sites, such as the testis in our case mirrors the behavior of related flaviviruses such as the Zika virus and other pestiviruses like BVDV and CSFV, which have been shown to persist in the male reproductive tract and are known to be shed via semen [36, 37, 41]. Persistent infection and immunological tolerance are well-documented in BVDV-infected bulls that acquire the virus *in utero*, resulting in long-term viral shedding in the absence of detectable antibodies [37, 40]. We found no histological evidence of inflammation in the testis, suggesting that the presence of APPV signal by ISH within the seminiferous tubules was not a consequence of a potential breach of the blood-testis barrier but rather due to immunological tolerance. Although the boar examined in our study was seronegative at the time of sampling, the presence of maternal antibodies early in life [13] complicates the interpretation of its immune status. The possibility of acquired immune tolerance cannot be ruled out and warrants further investigation.

The presence of viral RNA in the prostate and bulbourethral gland supports previous findings that identified APPV in boar semen [12, 13, 33]. A recent report described 98.9% identical APPV sequences detected in the serum of a CT-affected piglet, and in the semen dose used to inseminate the gilts in the farm, that strongly suggests semen-mediated transmission of the virus [34]. Given the widespread use of artificial insemination in commercial swine breeding, the potential for semen-borne transmission of APPV has considerable implications. Our findings highlight the necessity of rigorous monitoring of boar studs to prevent dissemination of the virus through breeding programs. Although APPV genome was not detected in 25 semen samples collected from two boar studs in Hungary (unpublished data), the limited sample size precludes firm conclusions, and continuous surveillance remains essential.

Three hypotheses may explain the ability of APPV to infiltrate previously protected compartments in mature animals: [1] testicular maturation alters cellular susceptibility and barrier function, enabling viral entry; or [2] chronic infection over several months facilitates viral adaptation or local immune modulation, allowing passage across the blood-testis barrier, or [3] *in utero* exposure to the virus may result in persistent infection, leading to long-term viral shedding across multiple organs, similar to what has been described for BVDV. These mechanisms are not mutually exclusive and merit further exploration using larger animal cohorts and time-course studies.

We have identified viral nucleic acid in lymphoid tissues and neurons, consistent with prior reports [6, 27, 31]. Detection in the periarteriolar lymphoid sheaths of the spleen, ileal GALT, and undifferentiated crypt enterocytes of the 6-month-old animal highlights systemic viral persistence. The presence of APPV in cerebellar and spinal neurons, even in an animal that had clinically recovered from CT, suggests low-level persistence in the central nervous system, as described in other congenital pestivirus infections [29].

In the present study, viral RNA was detected in the *zona glomerulosa* and, to a lesser extent, in the *zona fasciculata* of the adrenal cortex. This observation is consistent with findings on a recently described pestivirus of whale species, *Phocoena pestivirus*, in which viral RNA was detected in endocrine cells of the adrenal gland (as well as all other investigated organs) by qRT-PCR and ISH [43]. Similarly, the Zika virus was identified in adrenal tissue of a congenitally infected newborn by qRT-PCR, although not confirmed by immunohistochemistry [44]. In alpacas persistently infected with *Pestivirus bovis/tauri*, viral antigen was most prominent in the *zona fasciculata*, although all adrenal cell types were affected [45].

Pancreatic acinar cells and the endocrine cells of the islets of Langerhans have previously been identified as targets of *Phocoena pestivirus* infection [43]. In alpacas persistently infected with *Pestivirus bovis/tauri*, antigen was detected in acinar cells, stromal tissue, and the endocrine pancreas [45]. Similarly, in our study, viral nucleic acid was detected in the exocrine acinar cells, suggesting that the pancreas may represent a relevant site of pestivirus replication in pigs.

To corroborate these findings, FFPE extraction was performed and the presence of APPV was confirmed by qRT-PCR, indicating that cells of the adrenal gland and pancreas are susceptible to infection.

The main limitation of our study is that it involves only a single animal, which constrains the wider interpretation of the findings. However, to our knowledge there is no other report of a CT-affected piglet that has been kept and followed until sexual maturation. Furthermore, while RNAscope provides in situ detection of viral nucleic acid, it cannot confirm viral replication or infectious potential. Nonetheless, when considered alongside qRT-PCR data and previous challenge trials [12, 27], our findings provide compelling evidence for the role of the reproductive tract as a reservoir for APPV and source of shedding in the semen.

## Conclusion

We detected the virus in multiple organs using RNA in situ hybridization and qRT-PCR. Notably, positive signals were observed in the adrenal gland, the pancreas, and in cells located at and beyond the blood-testis barrier. Based on these findings, we strongly recommend screening boar studs for the presence of the virus, a measure that we have already initiated in our ongoing research. Furthermore, it is important to emphasize that boars born with congenital tremor, even if they subsequently recover, should not be kept for breeding purposes.

## Data Availability

Regarding the experiment, all data is presented in this publication.
